# Increased platelet distribution width and reduced IL-2 and IL-12 are associated with thrombocytopenia in *Plasmodium vivax* malaria

**DOI:** 10.1590/0074-02760200080

**Published:** 2020-07-17

**Authors:** Allyson Guimarães Costa, Yury Oliveira Chaves, Andréa Teixeira-Carvalho, Rajendranath Ramasawmy, Lis Ribeiro Valle Antonelli, Lucas Barbosa, Antonio Balieiro, Wuelton Marcelo Monteiro, Maria Paula Mourão, Marcus Vinicius Guimarães Lacerda, Olindo Assis Martins-Filho, Fabio Trindade Maranhão Costa, Adriana Malheiro, Paulo Afonso Nogueira

**Affiliations:** 1 Universidade do Estado do Amazonas, Programa de Pós-Graduação em Medicina Tropical, Manaus, AM, Brasil; 2 Fundação de Medicina Tropical Dr Heitor Vieira Dourado, Instituto de Pesquisa Clínica Carlos Borborema, Manaus, AM, Brasil; 3 Fundação Hospitalar de Hematologia e Hemoterapia do Amazonas, Diretoria de Ensino e Pesquisa, Manaus, AM, Brasil; 4 Universidade do Estado do Amazonas, Programa de Pós-Graduação em Ciências Aplicadas à Hematologia, Manaus, AM, Brasil; 5 Universidade Federal do Amazonas, Programa de Pós-Graduação em Imunologia Básica e Aplicada, Manaus, AM, Brasil; 6 Fundação Oswaldo Cruz-Fiocruz, Instituto Leônidas e Maria Deane, Programa de Pós-Graduação em Biologia da Relação Patógeno-Hospedeiro, Manaus, AM, Brasil; 7 Fundação Oswaldo Cruz-Fiocruz, Instituto Oswaldo Cruz, Programa de Pós-Graduação em Biologia Parasitária, Rio de Janeiro, RJ, Brasil; 8 Fundação Oswaldo Cruz-Fiocruz, Centro de Pesquisas René Rachou, Grupo Integrado de Pesquisas em Biomarcadores, Belo Horizonte, MG, Brasil; 9 Universidade Nilton Lins, Faculdade de Medicina, Manaus, AM, Brasil; 10 Fundação Oswaldo Cruz-Fiocruz, Instituto René Rachou, Laboratório de Biologia e Imunologia de Doenças Infecciosas e Parasitárias, Belo Horizonte, MG, Brasil; 11 Universidade Estadual de Campinas, Instituto de Biologia, Campinas, SP, Brasil

**Keywords:** platelet, Plasmodium vivax, IL-12p70, IL-2, IL-1 beta, thrombocytopenia

## Abstract

**BACKGROUND:**

Thrombocytopenia in malaria involves platelet destruction and consumption; however, the cellular response underlying this phenomenon has still not been elucidated.

**OBJECTIVE:**

To find associations between platelet indices and unbalanced Th1/Th2/Th17 cytokines as a response to thrombocytopenia in *Plasmodium vivax* infected (Pv-MAL) patients.

**METHODS:**

Platelet counts and quantification of Th1/Th2/Th17 cytokine levels were compared in 77 patients with uncomplicated *P. vivax* malaria and 37 healthy donors from the same area (endemic control group - ENCG).

**FINDINGS:**

Thrombocytopenia was the main manifestation in 55 patients, but was not associated with parasitaemia. The Pv-MAL patients showed increases in the mean platelet volume (MPV), which may be consistent with larger or megaplatelets. Contrary to the findings regarding the endemic control group, MPV and platelet distribution width (PDW) did not show an inverse correlation, due the increase in the heterogeneity of platelet width. In addition, the Pv-MAL patients presented increased IL-1β and reduced IL-12p70 and IL-2 serum concentrations. Furthermore, the reduction of these cytokines was associated with PDW values.

**MAIN CONCLUSIONS:**

Our data demonstrate that an increase in MPV and the association between reductions of IL-2 and IL-12 and PDW values may be an immune response to thrombocytopenia in uncomplicated *P. vivax* malaria.

Thrombocytopenia is one of the most frequent haematological alterations in acute malaria infections involving *Plasmodium falciparum* and *Plasmodium vivax* malaria (Pv-MAL).^1^ Thrombocytopenia is prominent in patients with splenomegaly and can be found in patients whose spleen is not palpable.^2^ ^,^ ^3^ Thrombocytopenia is not listed as a criterion for severe malaria, however, its clinical importance has been widely recognised when accompanied by multiple organ failures.^4^ ^,^ ^5^ ^,^ ^6^ The mortality rate in vivax malaria patients with severe thrombocytopenia alone can be comparable to that of falciparum malaria when associated with other danger signs of malaria (such as seizures, jaundice, bleeding, among others), and these can serve as red flags in order to promptly identify patients who may have severe malaria.^4^ ^,^ ^6^ ^,^ ^7^ ^,^ ^8^

The pathogenesis of thrombocytopenia caused by malaria involves multifactorial phenomenon and leads to destruction and consumption of platelets.^9^ In particular, in falciparum malaria, platelets also bind diffusely in systemic microvasculature, rather than pooling in the liver or spleen.^10^ ^,^ ^11^ Moreover, other pathophysiological mechanisms have recently been associated with thrombocytopenia caused by malaria, such as coagulopathy, splenic sequestration of damaged platelets, platelet aggregation, antibody-mediated platelet destruction and oxidative stress aggregation formation and dysmegakaryopoiesis.^4^ ^,^ ^12^ ^,^ ^13^ In Pv-MAL, thrombocytopenia is also common, however its pathogenesis is less well-known.

Platelets are small disc-shaped cellular fragments with no nucleus, and which have regenerated continuously from megakaryocyte (MK). The MKs are large, polyploid cells that have lost their proliferative ability and have become progressively differentiated, creating an invaginated membrane system for platelet formation.^14^ The steady state megakaryopoiesis is responsible for releasing of approximately 100 × 10^9^ platelets per day and lifespan is 7-10 days. Under inflammatory and thrombocytopenia conditions, such as malarial infection, platelet lifespan is reduced to 2-3 days and MKs differentiate in order to replenish platelets.^15^ ^,^ ^16^

In general, thrombocytopenia in malaria is accompanied by both higher mean platelet volume (MPV) and larger platelet distribution width (PDW), which arise concomitantly with reduced platelet counts.^12^ ^,^ ^17^ The increase in platelet volume is a result of the release of larger platelets in the circulation, also described as giant or mega platelets.^4^ ^,^ ^13^ ^,^ ^18^ ^,^ ^19^ ^,^ ^20^ Mega platelets are larger than 5 μm in the smear and are found in the peripheral circulation when there is increase of megakaryocyte production, due to reduced platelet level in the body. Even if the presence of mega platelets is not identified in the smear, it is possible to detect the increase in platelet volume indirectly, by increasing the MPV. Mega platelets also have a greater amount of granules, which, consequently, increases their ability to adhere and aggregate.^19^ Mega platelets and increased MPV may indicate a compensatory premature release of platelets from the bone marrow in order to compensate for the lower absolute number of platelets in the periphery.^4^ ^,^ ^18^ ^,^ ^19^ ^,^ ^20^ According to other studies, the megakaryocytic lineage is apparently preserved in bone marrow and able to release mega platelets.^4^ ^,^ ^13^ ^,^ ^18^ Since the blood stream medullary response can be triggered by cytokines, our study assessed serum concentrations of Th1/Th2/Th17 cytokines, in order to characterise a profile of the immune response in thrombocytopenia caused by *P. vivax* infections.

## SUBJECTS AND METHODS

*Study area and population* - 77 Pv-MAL patients (diagnosed as confirmed cases via examination of the thick blood smear) were enrolled at the Tropical Medicine Institute in the municipality of Coari, Amazonas State, Brazil. Coari is located at latitude: -4.08488, Longitude: -63.1417; 4º5’6” South, 63º8’30” West, in an area with a high incidence of malaria. It is estimated that approximately 30% of its inhabitants are at risk of malaria (annual parasitological index > 50). *P. vivax* is responsible for 99.6% of cases of malaria.^21^ All the patients had classic malaria symptoms with no signs of severe or complicated malaria.

Parasitaemia levels were defined as low parasitaemia (≤ 500 parasites/mm^3^) or mild (> 500 parasites/mm^3^). Blood cells were examined using an ABX Micros 60 haematology analyser (Horiba). 37 healthy donors were included in the study to form an endemic control group (ENCG). These were health professionals who are residents of this area, who had no previous history of malaria and tested negative in two thick blood smear examinations which were performed at intervals of 15 days. When detected, thrombocytopenia was classified as being either mild (50-150,000/mm^3^) or severe (< 50,000/mm^3^).^22^

A detailed serological screening was performed for hepatitis B virus, hepatitis C virus and HIV (Arquitect i2000SR, Abbott Diagnostics), and supervised by the Blood Bank (HEMOAM). In addition, participants were also screened for dengue Virus by polymerase chain reaction (PCR). After malaria diagnosis, blood samples were collected in EDTA tubes (BD Biosciences, USA), and sent in thermal boxes (maintained at 4ºC) to the Molecular Biology Laboratory of the Federal University of Amazonas in Coari.

*Serum cytokine measurements* - Frozen plasma samples were sent on dry ice to the Core Flow Cytometry Facility at the HEMOAM blood bank for cytokine measurement with the cocktail kit (CBA-BD/Biosciences Pharmingen, USA). The cytokines IL-2, IL-4, IL-5, IL-6, IL-10, TNF, IFN-γ and IL-17A were measured according to manufacturer’s instructions, and IL-1β, IL-8 (CXCL-8) and IL-12 (IL-12p70) were measured by ELISA (BD OptEIA Set II human kit, BD Biosciences Pharmingen, USA).

*Statistical analysis and data presentation* - All data were analysed using GraphPad prism software version 5. The Th1/Th2/Th17 cytokines levels were separated into non-thrombocytopenic, mild and severe thrombocytopenia according to platelet counts. Reference intervals for the platelet parameters according to the recommended European Federation of Clinical Chemistry and Laboratory Medicine were used.^23^ In both analyses, groups were compared with the endemic control group, which represented the basal state of cytokine levels. The Mann Whitney test was used to compare platelet counts and cytokine levels of Pv-MAL patients with those of the ENCG. In addition, platelet counts were compared among patients of the malaria group and classified into low and mild parasitaemia. Spearman’s rank correlation coefficient was used to assess the relationship between MPV and PDW indexes with platelet counts for the malaria group and endemic control group. A multinomial model classified the assessed platelet indices (Platelet counts, MPV and PDW indices) and cytokines between patients of the malaria group and classified them as having either low or mild parasitaemia. Finally, linear regression was performed to assess the predictors of thrombocytopenia in Pv-MAL patients in relation to the ENCG, taking into account platelet count indices and cytokine serum concentrations. An asterisk (*) indicates a significance level of p < 0.05, (**) p < 0.005 and (***) p < 0.0005.

*Ethics* - All protocols and consent forms were approved by the Research Ethics Committee at HEMOAM, under approval No. #449.864/2011. Participants read and signed the written informed consent form prior to enrollment, according to Declaration of Helsinki and Resolution 466/12 of the National Health Council for research involving human subjects. All patients were treated according to recommendations of Brazilian Ministry of Health.

## RESULTS

All patients included in the study had uncomplicated paroxysms at the time of seeking medical care. None of the patients reported diarrhea or vomiting. 53 (68.8%) patients were male and 24 (31.2%) were female. 15 of the patients (19.5%) reported primary malaria and 62 reported at least one previous malaria infection. None of the patients showed signs of anaemia or severe malaria. 30 patients had leukopenia (38.9%) and lymphopenia was evident in 32 patients (41.5%).

**Fig. 1:**
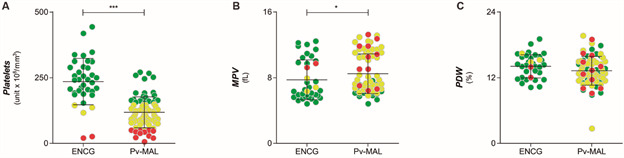
comparison between platelet counts in *Plasmodium vivax* malaria (Pv-MAL) patients and endemic control group (ENCG). (A) Comparison of platelet counts in ENCG and Pv-MAL patients. Thrombocytopenia was defined as a platelet count less than 150,000/mm^3^ and then classified as mild (50-150,000/mm^3^) and severe (< 50,000/mm^3^) thrombocytopenia. Patients were classified as non-thrombocytopenic when they had platelet counts above 150,000/mm^3^. The individuals were grouped according in non-thrombocytopenic (green circle), mild (yellow circle) and severe thrombocytopenia (red circle). (B) Comparison of mean platelet volume (MPV) in Pv-MAL patients and ENCG; (C) Comparison of platelet distribution width (PDW) in Pv-MAL patients and ENCG.

Thrombocytopenia was observed in 55 of 77 patients (71.4%), also in five of the 37 individuals in the ENCG. Thrombocytopenia was classified as being either moderate (51-149 x 10^3^ platelets/μL) or severe (below 50 x 10^3^ platelets/μL). Among the patients, 44 (80%) had moderate thrombocytopenia and 11 displayed severe thrombocytopenia (20%); none of the patients needed platelet transfusion. In Fig. 1A, coloured dots illustrate the Pv-MAL patients classified into non-thrombocytopenic, mild and severe thrombocytopenic individuals. The average age of the patients was 37.0 ± 14.3 years, the age of Pv-MAL patients did not affect platelet count according to Spearman’s rank correlation coefficient of 0.1373 (p-value = 0.2338). Among the 37 individuals used as the ENCG, three had moderate thrombocytopenia (8.1%) and two (5.4%) had severe thrombocytopenia and coloured as thrombocytopenia status. The average age of the patients was 29.4 ± 8.45 years in ENCG, though this did not influence the platelet count (r = 0.2365 and p-value = 0.0738).

**Fig. 2:**
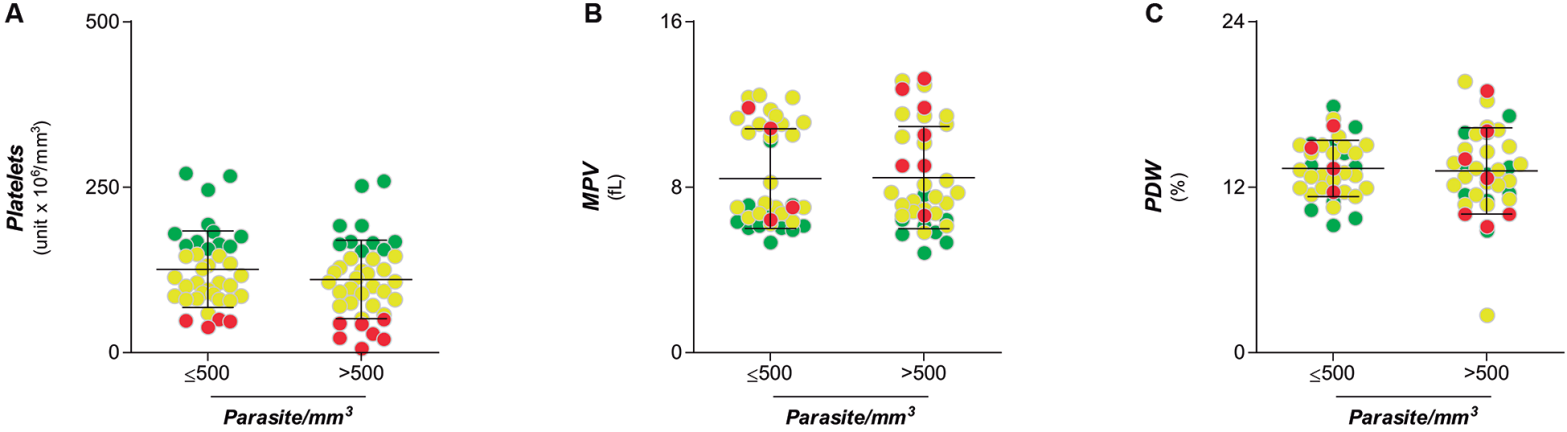
comparison of parasitaemia with platelet indices. (A) Comparison of platelet indices according to parasitaemia classified in ≤ 500 parasites/mm^3^ and 501-to-20,000 parasites/mm^3^. (B) Comparison of mean platelet volume (MPV) in relation to parasitaemia; (C) Comparison of platelet size heterogeneity (platelet distribution width - PDW) in relation to parasitaemia. The individuals were grouped according in non-thrombocytopenic (green circle), mild (yellow circle) and severe thrombocytopenia (red circle).

Platelet counts were used to assess whether the thrombocytopenia caused by vivax malaria was due to peripheral destruction or bone marrow disease. The average MPV index in Pv-MAL patients was higher than the average in the ENCG, although both were within normal range (Fig. 1B), and PDW values did not differ among the ENCG (Fig. 1C). No patient presented high parasitaemia. 38 patients (49.4%) had low parasitaemia, with parasite counts being below 500 parasites per milliliter (≤ 500 parasites/mm^3^), while 39 Pv-MAL patients (50.6%) showed moderate parasitaemia (between 501 to 20,000 parasites/mm^3^). However, not all platelet indices differed in relation to parasitaemia (Fig. 2A-C).

**Fig. 3:**
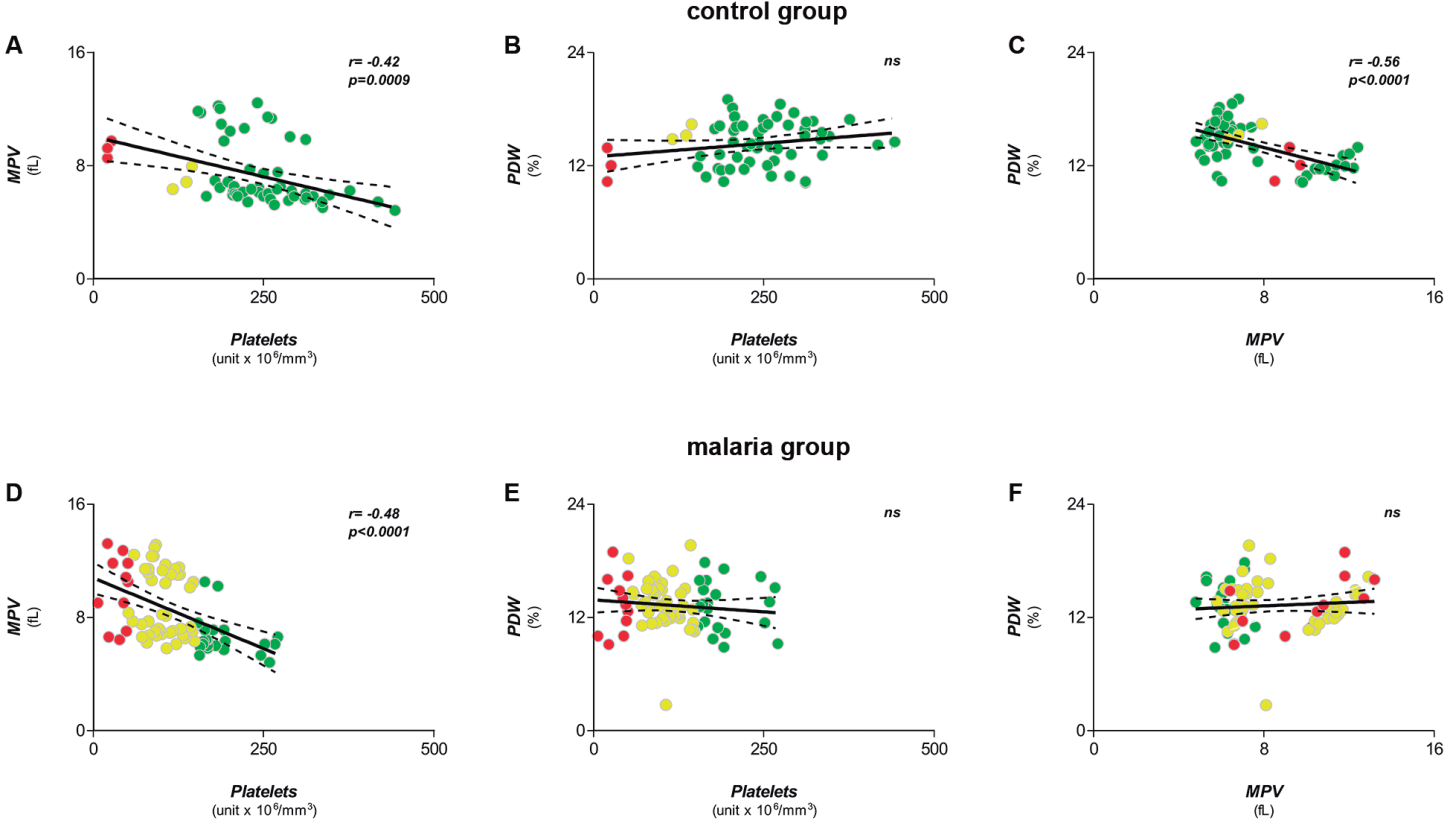
comparison of correlations among platelet indices between *Plasmodium vivax* malaria (Pv-MAL) patients and endemic control group (ENCG). (A-C) Correlations between mean platelet volume (MPV) and platelet counts, platelet distribution width (PDW) and platelet counts, PDW and MPV in ENCG. (D-F) Correlations between MPV and platelet counts, PDW and Platelet counts, PDW and MPV in Pv-MAL. The individuals were grouped according in non-thrombocytopenic (green circle), mild (yellow circle) and severe thrombocytopenia (red circle).

In general, lower platelet counts are associated with higher platelet volumes. Both groups showed an inverse correlation between mean platelet volume (MPV) and platelet counts (Fig. 3A-D), though not in relation to PDW values (Fig. 3B-E). It is also well known that PDW correlates with MPV in normal test subjects, and Fig. 3C-F shows this correlation was lost in Pv-MAL patients.

**Fig. 4:**
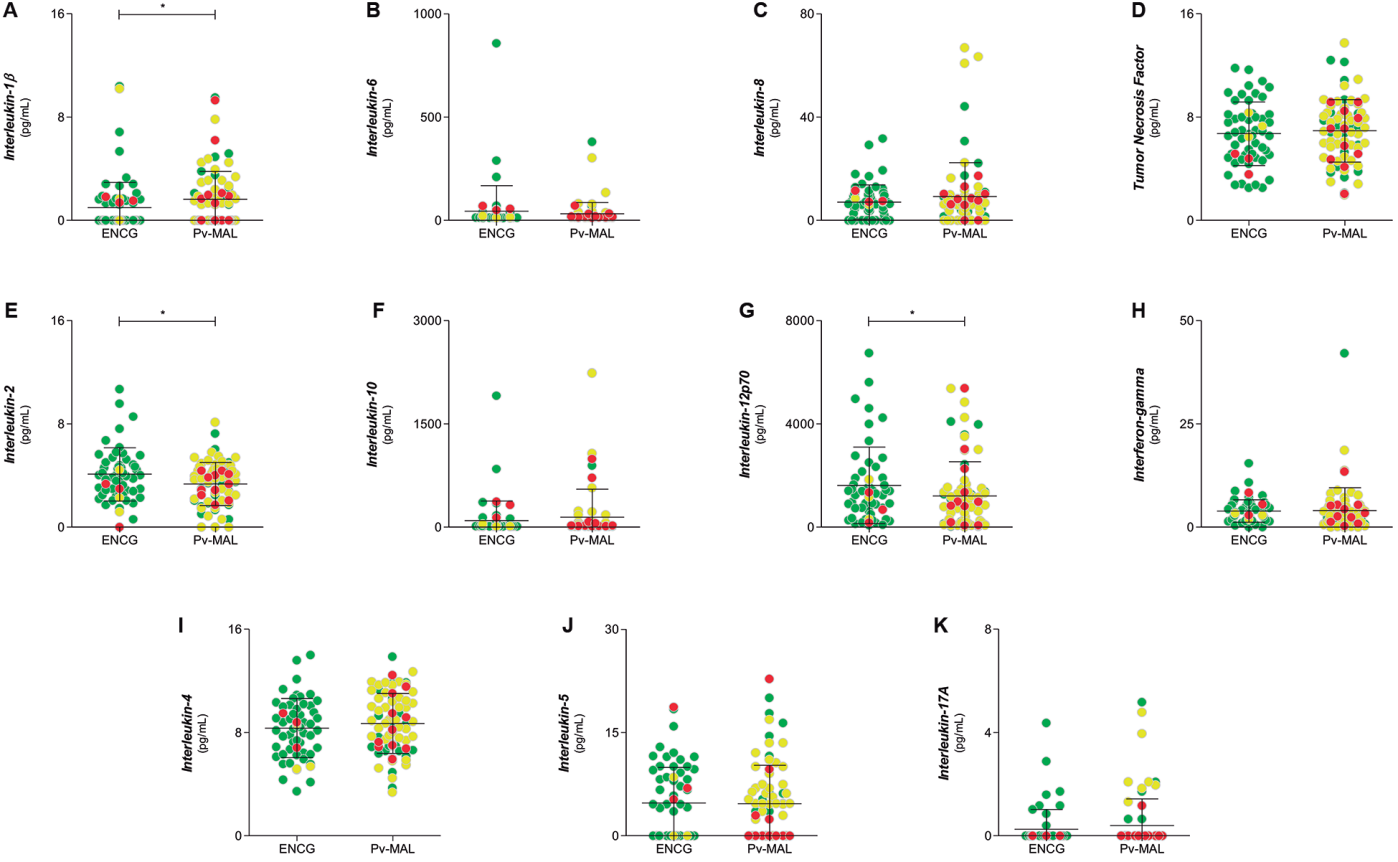
comparison of serum concentrations of Th1/Th2/Th17 cytokines in endemic control group (ENCG) and *Plasmodium vivax* malaria (Pv-MAL). The Th1/Th2/Th17 cytokines was dotted between ENCG and Pv-MAL patients. They were separated in a production order and serum concentrations, picogram per milliliter (pg/mL): (A) IL-1β; (B) IL-6; (C) IL-8; (D) TNF; (E) IL-2; (F) IL-10; (G) IL-12p70; (H) IFN-γ; (I) IL-4; (J) IL-5 and (K) IL-17A. The individuals were grouped according in non-thrombocytopenic (green circle), mild (yellow circle) and severe thrombocytopenia (red circle).

The serum concentrations of Th1/Th2/Th17 cytokines were compared in the Pv-MAL patients and the endemic control group (Fig. 4). Pv-MAL patients showed a reduction of IL-12p70 and IL-2 levels, while IL-1β was higher in relation to the ENCG (Fig. 4). Other cytokines showed no differences in the comparison between groups.

To assess predictors of thrombocytopenia using cytokine levels of Th1/Th2/Th17 and platelet indices, a multinomial model was used to exclude parasitaemia as a confounding factor (Table). For this, the serum levels of patients with mild parasitaemia were used as a reference group (Table). We observed that the crude odds ratio in the serum concentrations of IL-10, and IL-17A increased individually in response to mild parasitaemia. When Th1/Th2/Th17 cytokines and platelet indices were assessed together, the adjusted odds ratio for the serum concentration of IL-10 and IL-17A also increased in relation to the mild parasitaemia group.

Finally, a linear regression was performed to assess predictors of thrombocytopenia in Pv-MAL patients in relation to the ENCG. Since the PDW index showed negative correlation with platelet counts, we used it as a dependent variable, while the other platelet indices and cytokine levels were considered independent variables (Table). The linear regression showed that the levels of IL-1β, IL-2 and IL-12 were associated with altered PDW in Pv-MAL patients, and they may be predictors of the medullar response to thrombocytopenia caused by vivax malaria.

**TABLE:** Multivariate analyses of Th1/Th2/Th17 cytokines associated with parasitaemia (binomial) and platelet indices

Logistic regression of Pv-MAL patients in relation to parasitaemia				Linear regression predicting PDW between Pv-MAL patients and endemic controls		
Mild parasitaemia (Reference)	Crude OR (CI 95%)	Adjusted OR (CI 95%)	p (LR-test*)*	Patients (Reference)	Estimates (CI 95%)	p value
Platelets	1.00 (0.99, 1.00)	1.00 (0.99, 1.01)	0.510	Platelets	-0.01 (-0.01, 0.01)	0.315
MPV	1.01 (0.84, 1.21)	1.01 (0.75, 1.37)	0.947	MPV	-0.03 (-0.27, 0.20)	0.770
PDW	0.97 (0.82, 1.16)	0.99 (0.81, 1.21)	0.93	PDW	1.44 (0.24, 2.63)	0.019
IL-1β	1.02 (0.99, 1.06)	1.03 (0.99, 1.07)	0.100	IL-1β	-0.03 (-0.05, -0.01)	0.047
IL-6	1.00 (0.99,1.00)	1.00 (0.99, 1.0001)	0.123	IL-6	-0.01 (-0.01, 0.01)	0.446
IL-8	1.00 (0.99, 1.01)	0.99 (0.98, 1.00)	0.197	IL-8	-0.01 (-0.01, 0.01)	0.801
TNF	1.00 (0.99, 1.01)	1.01 (0.99, 1.03)	0.312	TNF	0.01 (-0.01, 0.01)	0.371
IL-2	1.00 (0.98, 1.01)	0.99 (0.96, 1.01)	0.301	IL-2	-0.02 (-0.04, -0.01)	0.030
IL-10	1.00 (1.00, 1.00)	1.00 (1.00, 1.00)	0.029	IL-10	-0.01 (-0.01, 0.01)	0.573
IL-12p70	1.00 (0.99, 1.00)	1 (0.99, 1.00)	0.692	IL-12p70	-0.01 (-0.01, -0.01)	0.038
IFN-γ	1.00 (0.99, 1.00)	0.99 (0.99, 1.01)	0.900	IFN-γ	-0.01 (-0.01, 0.01)	0.413
IL-4	0.99 (0.99, 1.00)	0.99 (0.98, 1.00)	0.122	IL-4	0.01 (-0.01, 0.01)	0.833
IL-5	1.00 (0.96,1.04)	0.99 (0.94, 1.04)	0.773	IL-5	0.04 (-0.01, 0.08)	0.052
IL-17A	1.02 (1.00, 1.05)	1.03 (1.00, 1.07)	0.043	IL-17A	-0.01 (-0.03, 0.02)	0.712

CI: confidence interval; MPV: mean platelet volume; OR: odds ratio; PDW: platelet distribution width; Pv-MAL: *Plasmodium vivax* malaria.

## DISCUSSION

Thrombocytopenia is the most common haematological disorder in patients with malaria and one of the major concerns when accompanied by multiple organ failures.^5^ ^,^ ^6^ In our study, thrombocytopenia was the main manifestation among patients with vivax malaria, though the reduction in platelet count was not associated with parasitaemia. Although some patients presented severe thrombocytopenia, none of them presented bleeding. Very low platelet counts during malaria are considered transient, for those who do not present bleeding, platelet transfusions are unnecessary.^24^ Platelet function is compromised in thrombocytopenia caused by malaria, and this is generally evidenced by changes in its parameters.^18^ Here, the Pv-MAL patients displayed an increased MPV that may indirectly indicate the presence of mega platelets.^4^ A negative correlation between platelet counts and MPV can be observed, but this inverse correlation is not restricted to malaria, since it is usually found in other physiological conditions.^4^ ^,^ ^18^ ^,^ ^24^ Furthermore, we observed that an inverse correlation between MPV and PDW in the endemic control group was lost in Pv-MAL patients due a trend of increases in PDW and MPV.

The total platelet mass is considered as a product of platelet count X MPV, and this inverse correlation may be closely regulated.^23^ Cytokine levels in malaria have dual effects in the pathogenesis of, and protection against, malaria.^25^ The cytokine imbalance in thrombocytopenia caused by vivax malaria has been reported by several studies without a consensus about which cytokines could be associated to thrombocytopenia.^4^ ^,^ ^13^ ^,^ ^20^ ^,^ ^26^ ^,^ ^27^ In this study, we assessed immune responses in relation to other parameters as well as platelet counts. Of all the cytokines, only IL-1β, IL-12 and IL-2 differed in Pv-MAL patients, and were associated with increases in the PDW values. Thus, the association of PDW with these differences may be considered a reduction of Th1 responses and thus the main mechanism which induces the elevation of MPV and PDW, in accordance with the findings of other authors.^18^

Furthermore, parasitaemia was associated with higher levels of IL-10 and a slight increase in the serum concentration of IL17A. Increased levels of IL-10 have already been seen in patients with higher parasitaemia and patients with recurrent malaria.^28^ ^,^ ^29^ Here, this imbalance in IL-10 and IL17A seems to be a shift from a pro-inflammatory response to an anti-inflammatory response, which explains the low frequencies of severe malarial disease among individuals living in endemic areas.^29^

This study had several limitations. The sample size of patients was small and PCR for malaria diagnoses was not performed in all of those enrolled in this study. Nor did we evaluate antimicrobial failure. The population of the municipality of Coari is very well-informed about malaria and knows about the need to diagnose malaria at the onset of the disease, hence, this may explain why all the patients had uncomplicated malaria. Another limitation to the study was the semi-quantification of parasites which is carried out in the Brazilian Amazon, and which made it impossible to assess further correlations, as also observed by a few other studies.^18^ ^,^ ^30^ A further limitation to this study was that documentation of mega platelets was not done at the time of blood collection, however, the aim of the study was to assess changes in the immune response associated with thrombocytopenia. It is likely that the changes in MPV and PDW observed here may be due the release of the larger platelets also called as mega platelets or giant platelets.^4^ ^,^ ^19^ These mega platelets may be detected indirectly by the increase in platelet volume in thrombocytopenia caused by malaria. The greater amount of α-granules and dense granules enhance the mega platelets’ ability of to adhere and aggregate with small platelets, as observed in peripheral blood smears of children infected with *P. falciparum* malaria.^19^ These increases provide equal or similar primary haemostasis and may be the reason why bleeding episodes are rare in acute malaria infection, despite the thrombocytopenia.^4^ ^,^ ^19^ Thus, the reason for our assumption is that megakaryocytic lineage is apparently preserved in bone marrow and able to release mega platelets in the blood stream.^4^ ^,^ ^13^ ^,^ ^18^ Our data encourage studies that assess the release of mega platelets in thrombocytopenia caused by *P. vivax* infections.

In conclusion, the increase in MPV indirectly indicated the presence of mega platelets. The loss of its correlation with PDW in Pv-MAL patients may also be a consequence of the release of mega platelets. The reduction of IL-2 and IL-12 associated with PDW may be consistent with regulation of immune response which is associated with appearance of mega platelets in thrombocytopenia caused by vivax malaria.
